# Optimization of Tilmicosin-Loaded Nanostructured Lipid Carriers Using Orthogonal Design for Overcoming Oral Administration Obstacle

**DOI:** 10.3390/pharmaceutics13030303

**Published:** 2021-02-25

**Authors:** Jia Wen, Xiuge Gao, Qian Zhang, Benazir Sahito, Hongbin Si, Gonghe Li, Qi Ding, Wenda Wu, Eugenie Nepovimova, Shanxiang Jiang, Liping Wang, Kamil Kuca, Dawei Guo

**Affiliations:** 1 Center for Veterinary Drug Research and Evaluation, MOE Joint International Research Laboratory of Animal Health and Food Safety, College of Veterinary Medicine, Nanjing Agricultural University, 1 Weigang, Nanjing 210095, China; 2018107005@stu.njau.edu.cn (J.W.); vetgao@njau.edu.cn (X.G.); 2016107022@njau.edu.cn (Q.Z.); 2015207038@njau.edu.cn (B.S.); jiangshanxiang@163.com (S.J.); wlp71@njau.edu.cn (L.W.); 2 College of Animal Science and Technology, Guangxi University, 100 Daxuedong Road, Nanning 530004, China; shb2009@gxu.edu.cn (H.S.); lgh2005@gxu.edu.cn (G.L.); 3 School of Pharmacy, Bengbu Medical College, 2600 Donghai Avenue, Bengbu 233030, China; 2019003@bbmc.edu.cn; 4 Department of Chemistry, Faculty of Science, University of Hradec Kralove, 50003 Hradec Kralove, Czech Republic; eugenie.nepovimova@uhk.cz

**Keywords:** tilmicosin, nanostructured lipid carriers, orthogonal design, intestinal absorption, MDCK-chAbcg2/Abcb1 cell monolayer

## Abstract

Tilmicosin (TMS) is widely used to treat bacterial infections in veterinary medicine, but the clinical effect is limited by its poor solubility, bitterness, gastric instability, and intestinal efflux transport. Nanostructured lipid carriers (NLCs) are nowadays considered to be a promising vector of therapeutic drugs for oral administration. In this study, an orthogonal experimental design was applied for optimizing TMS-loaded NLCs (TMS-NLCs). The ratios of emulsifier to mixed lipids, stearic acid to oleic acid, drugs to mixed lipids, and cold water to hot emulsion were selected as the independent variables, while the hydrodynamic diameter (HD), drug loading (DL), and entrapment efficiency (EE) were the chosen responses. The optimized TMS-NLCs had a small HD, high DL, and EE of 276.85 ± 2.62 nm, 9.14 ± 0.04%, and 92.92 ± 0.42%, respectively. In addition, a low polydispersity index (0.231 ± 0.001) and high negative zeta potential (−31.10 ± 0.00 mV) indicated the excellent stability, which was further demonstrated by uniformly dispersed spherical nanoparticles under transmission electron microscopy. TMS-NLCs exhibited a slow and sustained release behavior in both simulated gastric juice and intestinal fluid. Furthermore, MDCK-chAbcg2/Abcb1 cell monolayers were successfully established to evaluate their absorption efficiency and potential mechanism. The results of biodirectional transport showed that TMS-NLCs could enhance the cellular uptake and inhibit the efflux function of drug transporters against TMS in MDCK-chAbcg2/Abcb1 cells. Moreover, the data revealed that TMS-NLCs could enter the cells mainly via the caveolae/lipid raft-mediated endocytosis and partially via macropinocytosis. Furthermore, TMS-NLCs showed the same antibacterial activity as free TMS. Taken together, the optimized NLCs were the promising oral delivery carrier for overcoming oral administration obstacle of TMS.

## 1. Introduction

Nanostructured lipid carriers (NLCs), the second-generation lipid-based nano drug delivery system, are derived from an admixture of solid lipid and liquid lipid and regarded as an effective oral carrier in virtue of their possibility to increase the solubility and oral bioavailability of poorly water-soluble drugs [1,2,3]. NLCs exhibit higher drug loading and lower drug expulsion during storage than solid lipid nanoparticles (SLNs) because of their imperfect binary-lipid particle matrix [4,5,6]. In addition, it is found that NLCs present good biocompatibility and stability, which is also available for large-scale production [7,8].

Tilmicosin (TMS) is a semi-synthetic macrolide antibiotic widely used in veterinary medicine with properties including broad antimicrobial spectrum, low inhibitory concentration, a large distribution volume, and long elimination half-life [9]. TMS is recommended to treat porcine bacterial respiratory diseases associated with *Mycoplasma*, *Streptococcus*, *Pasteurella haemolytica*, *Pasteurella multocida*, *Actinobacillus pleuropneumoniae,* and *Staphylococcus* [10,11,12]. It is easy to cause an acute cardiotoxic effect when administered by injection owing to its transient high drug concentration [13]; hence, oral administration would be a more suitable delivery method for TMS, but this administration route suffers from problems such as poor water solubility, sensitivity to gastric acid, bitterness, and low bioavailability [14]. Hence, it is reported SLNs [13], silica nanoparticles [15], alginate–chitosan nanogels [16] and polymeric nanoparticle [17] were used to load TMS, indicating that a stable, safe, and efficient delivery system should be developed for TMS.

In our previous studies, three TMS-loaded NLCs (TMS-NLCs) formulations with different solid lipid were prepared that could improve oral bioavailability compared to the marketed formulation in broilers [18]. Furthermore, it was proved that TMS-NLCs were an effective formulation with excellent drug loading, encapsulation efficiency, and stability, which enhanced the transcellular transport across Caco-2 cells and escaped the lysosome degradation [19]. An orthogonal experimental design is broadly applied in many fields to investigate the relative importance of each factor and identify the ideal values for various factors [20]. In this study, an orthogonal experimental design was utilized to evaluate the effect of different factors on TMS-NLCs with smaller hydrodynamic diameter (HD), high drug loading (DL), and uniform dispersion. The Madin–Darby canine kidney (MDCK) cell line was used as an epithelial cell model in this study, because it is widely adopted to simulate the gastrointestinal tract and can differentiate into columnar epithelium with tight junctions and form cell monolayers in 5–7 days. However, it is not suitable for studying the absorption mechanism involved in transporter regulation due to its relatively negligible expression of endogenous transporters. Hence, it has been widely used for overexpressing exogenous genes. MDCK-chAbcg2/abcb1 cells, unlike Caco-2 cells only expressing P-glycoprotein (P-gp) used in our previous study, overexpressing both P-gp and Breast Cancer Resistance Protein (BCRP) [21], were utilized to evaluate the permeability of TMS-NLCs and explore its absorption mechanism.

## 2. Materials and Methods

### 2.1. Materials

Tilmicosin (TMS, 91.4% *w*/*w*) was obtained from Ningxia Teirui Pharmaceutical Corporation (Yinchuan, China). Poloxamer 188 (P188, ≥99.0% *w*/*w*) was supplied by Shanghai Yunhong Chemical Co., Ltd. (Shanghai, China). Oleic acid (OA), stearic acid (SA, 40% *w*/*w*), Tween 80 (T80), verapamil hydrochloride (99%), chlorpromazine hydrochloride (CHI, 98%), and propranolol hydrochloride (98%) were obtained from Aladdin (China). Dynasore (>99%) was purchased from Abcam (London, UK). Methyl-β-cyclodextrin (MβCD) was provided from MACKLIN (Shanghai, China). 3-(4,5-dimethyl-2-thiazolyl)-2,5-diphenyl-2-H-tetrazolium bromide (MTT, 98%) and lucifer yellow were purchased from Sigma Aldrich (St. Louis, MO, USA). 5-(*N*-ethyl-*N*-isopropyl)-amiloride (EIPA) was bought from ApexBio (Houston, TX, USA).

### 2.2. Formulation Optimization of TMS-NLCs

#### 2.2.1. Preparation of TMS-NLCs

TMS-NLCs were fabricated using the high shear-ultrasound method reported previously [19]. Briefly, 10 mL of aqueous phase containing P188 and 1 g mixture of SA and OA including Tween 80 and TMS was heated up to 80 °C (the melting point of SA is 69–72 °C), respectively. Then, the lipid phase was promptly transferred into the aqueous phase under magnetic stirring at 600 rpm. Subsequently, the mixture was homogenized for 5 min at 11,000 rpm with a High Shear Dispersion Emulsifying Machine (FM200, IKA, Staufen, Germany), and treated by probe sonication for 20 min at 300 W with an Ultrasonic Cell Disruptor (JY96-II, Scientz, Ningbo, China). The emulsion was kept in an 80 °C water bath for the above process. The obtained emulsion was dispersed into 11 mL of ice water with homogenization in 60 s and kept in an ice bath for 10 min to solidify the particles and obtain TMS-NLCs suspensions. 

#### 2.2.2. Orthogonal Experimental Design

The solubility of tilmicosin in different lipids and the appropriate emulsifiers has successfully screened to prepare TMS-NLCs in our previous study [18]. This study aimed to further optimize TMS-NLCs using an orthogonal experiment design. Single-factor experiments were firstly performed to determine the effect of preparation composition and process conditions on TMS-NLCs, including emulsifiers to SA to OA ratio, mixed lipids ratio, drug to mixed lipid ratio, cold water to hot emulsion ratio, ultrasonic time, and cold water dispersion time. Based on the results of single-factor experiments, the ratios of SA to OA (A), emulsifier to mixed lipids (B), drug to mixed lipids (C), and cold water to hot emulsion (D) were the major contributing factors in the preparation of TMS-NLCs (Supplementary Materials, Tables S1–S6). Consequently, these parameters were investigated in this study using an orthogonal table L_9_ (3^4^) designed by the software, Orthogonal Designing Assistant II (Sharetop Software Studio, Version 3.1, Beijing Qingsi Technology Co., Ltd, Beijing, China). Herein, L represented the orthogonal table; 9 was the total number of experiments; 3 was the number of levels of various factors; 4 represented the maximum allowed number of factors. The hydrodynamic diameter (HD), entrapment efficiency (EE), and drug loading (DL) were the chosen responses. Using an orthogonal L_9_ (3^4^) design (Table 1), the purpose was to investigate the impact of 4 factors mentioned above on HD, EE, and DL, and to establish the optimal prescription component and preparation process.

### 2.3. Characterization of TMS-NLCs

#### 2.3.1. Hydrodynamic Diameters, Polydispersity Index, and Zeta Potentials of TMS-NLCs

Malvern Zetasizer (Nano ZSE, Malvern Instruments, Worcestershire, UK) was used to measure the mean HD, polydispersity index (PDI), and zeta potential (ZP) of TMS-NLCs by dynamic light scattering (DLS) at an angle of 90° and equilibration time of 120 s at 25 °C. The samples were diluted 100 times with the distilled water prior to analysis. 

#### 2.3.2. The Morphology of TMS-NLCs

Transmission electron microscopy (TEM; Tecnai 12, Philips, Amsterdam, The Netherlands) was used to observe the morphology of TMS-NLCs. TMS-NLCs emulsion was firstly diluted and then dropped on 300 mesh copper grids. After drying, 2% (*w*/*v*) phosphotungstic acid solution was utilized to negatively stain the samples for 2 min. Then, the samples were dried for TEM analysis.

#### 2.3.3. Entrapment Efficiency and Drug Loading of TMS-NLCs

The EE and DL of TMS-NLCs was determined by the ultrafiltration centrifugation method [22]. Free TMS was separated from TMS-NLCs by an ultrafiltration centrifugal tube (MWCO: 100 kDa, Millipore, Bilrika, MA, USA). Briefly, 0.5 mL 1:10 diluent was put into the inner chamber and then centrifuged for 20 min at 5000 rpm in 4 °C. The filtrate in the outer chamber was collected, constant-volumed to 1 mL and analyzed by high-performance liquid chromatography (HPLC), which was represented as the amounts of free drug (W_free_). The EE and DL were calculated by the following equations:

EE% = (W_total_ − W_free_) × 100%/W_total_

DL% = (W_total_ − W_free_) × 100%/W_NLCs_

where W_total_, W_free_, and W_NLCs_ were the amounts of total TMS, the free TMS, and NLCs, respectively.

#### 2.3.4. In Vitro Drug Release of TMS-NLCs

The in vitro drug release behavior of TMS-NLCs was carried out over 48 h by the dialysis bag method. Briefly, TMS-NLCs or the corresponding active pharmaceutical ingredient (API) of TMS (3 mL, 10 mg/mL) was added into a dialysis bag (MWCO of 100 kD, 16 mm, and 0.79 mL/cm), immersed in the simulated gastric or intestinal juice (500 mL) at 37 °C, and stirred under the magnetic stirrer with 100 rpm. At the set time, the samples (1 mL) were taken out and substituted by the same volume of fresh receptor fluids, and the TMS concentrations were detected by HPLC.

#### 2.3.5. High-Speed Centrifugal Stability

For the stability studies, TMS-NLCs were centrifuged at 10,000 rpm for 5, 10, 20, and 30 min at 4 and 25 °C, respectively. The HD, PDI, and ZP of supernatant were measured. The stability constant (K_E_) was calculated according to the following equation.

K_E_ = |R_0_ − R|/R_0_

R_0_ is HD before centrifugation, and R is HD after centrifugation.

#### 2.3.6. HPLC Analysis of TMS

In this study, the concentrations of TMS in various media were detected with HPLC (Thermo U3000, Thermo Fisher Scientific, New York, NY, USA) with a C18 Reverse-phase column (5 µm, 4.6 mm inner diameter × 250 mm length, Agilent, Palo Alto, CA, USA). The mobile phase was acetonitrile–tetrahydrofuran–water–n-dibutylamine phosphate buffer salt solution (12:5.5:80:2.5; *v*/*v*). The column temperature was 30 °C, the injection volume was 20 µL, the UV detection wavelength was 291 nm, and the flow rate was 1 mL/min. The samples were filtered through a 0.22 µm filter membrane before HPLC analysis. The concentration of TMS was calculated using the standard curve equation (*y* = 31.314*x* − 2.0603, R^2^ = 0.9999) for HPLC analysis of TMS, which had the good linear pattern over a range from 1 to 20 µg/mL as reported previously [17].

### 2.4. Validation of Cell Monolayer Models

#### 2.4.1. The Culture of Cell Monolayers

MDCK and MDCK-chAbcg2/Abcb1 cells were seeded on 12-well Transwell^®^ polycarbonate inserts at a density of 2 × 10^5^ cells per well to constitute in vitro cell monolayer models. The apical side (AP) of the insert was filled with 0.5 mL medium, and the basolateral (BL) side was filled with 1.5 mL medium. The medium was replaced every two days. It took 5~7 days to form an intact cell monolayer.

#### 2.4.2. Integrity of Cell Monolayers

During the cultivation, the integrity of cell monolayers was evaluated by the transepithelial electrical resistance (TEER) values measured with Epithelial Volt-Ohm Meter (Millipore, Burlington, MA, USA). Monolayers with TEER values over 500 and 1700 Ω·cm^2^ of MDCK and MDCK-chAbcg2/Abcb1 cells respectively were used for drug transport experiments, and the TEER values were also checked after the transport experiment to guarantee the integrity of cell monolayers [23].

TEER = (R_total_ − R_blank_) × A (Ω·cm^2^)

R_total_ was the measured resistance values, R_blank_ was the resistance of insert membranes without cells, and A was the surface area of transwell that was 1.12 cm^2^.

#### 2.4.3. Transmission Electron Microscopy of Monolayers

TEM was used to observe the morphology and appearance of monolayers. After 7 days’ culture, monolayers were washed with PBS twice, followed by fixation with 2.5% glutaraldehyde overnight and 1% osmium acid for 2 h; then, they were dehydrated with gradient concentrations of ethanol and embedded in araldite resin at 60 °C for 48 h. After that, the samples were cut and stained with uranyl acetate and lead citrate for 10 min, respectively, and finally observed with TEM.

#### 2.4.4. Permeability of Cell Monolayers

After a week of cultivation, the AP side was filled with 0.5 mL propranolol (100 µM) or lucifer yellow (225 µM) and 1.5 mL of Hank’s solution on the BL side for 4 h at 37 °C. The solution on the BL side was collected for fluorescence analysis. The detection wavelengths were 428/540 nm (fluorescein) and 290/330 nm (propranolol), respectively. The apparent permeability coefficient (P_app_) was calculated according to the following equation [24]:

P_app_ = dQ/(dt * AC_0_)

where dQ/dt is the transport rate in the receiver solution, A is the membrane surface area, and C_0_ is the initial concentration in the donor compartment.

### 2.5. Effect of NLCs and Endocytosis Inhibitors on Cell Viability

MTT assay was used to evaluate cell viability. Cells were seeded on 96-well plates 1 × 10^4^ cells per well, cultured for 24 h until 90% confluent, and exposed to different concentrations of NLCs for 4 h. Then, the medium was replaced with 20 µL MTT (5 mg/mL) and 180 µL serum-free medium. After incubation for 4 h, the medium containing MTT was substituted with 150 µL DMSO. The absorbance of formazan was measured by a microplate reader (BIO-RAD, Hercules, CA, USA) at 490 nm. Untreated cells were taken as the control. The cytotoxicity was expressed as the cell viability compared with the untreated cells. Similarly, the cytotoxicity of endocytosis inhibitors were also evaluated, and the concentrations of each drug were chosen on the basis of the published literature [25], and EIPA (50 µM), chlorpromazine (30 µM), dynasore (80 µM), and MβCD (2 mM) were used in the present study.

### 2.6. The Permeability of TMS-NLCs across MDCK-chAbcg2/Abcb1 Cell Monolayers

The permeability efficiency was evaluated by comparing the bidirectional transport between API and TMS-NLCs. Briefly, apical-to-basolateral (AP→BL) and basolateral-to-apical (BL→AP) transport across monolayers was conducted by adding 10 µg/mL API or TMS-NLCs to donor compartments and incubating at 37 °C for 4 h. Then, the samples from receiver compartments were taken to analyze TMS concentrations by HPLC.

TMS is the substrate of glycoprotein (P-gp) [26]. To evaluate the inhibition effect of TMS-NLCs on P-gp mediated efflux, cell monolayers were incubated with the P-gp inhibitor verapamil (100 µM) for 1 h, and then, the medium was replaced with a mixture of verapamil and API or TMS-NLCs on one side and co-incubated for 4 h for bidirectional transport assay. 

The efflux ratio (ER) and net efflux ratio (NER) values were obtained from the following equation [27,28]:

ER = P_app_(BL→AP)/P_app_(AP→BL)

NER = ER(MDCK-chAbcg2/Abcb1)/ER(MDCK).

### 2.7. Transport Mechanism of TMS-NLCs across MDCK-chAbcg2/abcb1 Monolayers

To investigate the transport mechanism of TMS-NLCs, propranolol (100 µM) and lucifer yellow (225 µM) as the tracers were added to the AP side and co-incubated with TMS-NLCs for 4 h. The tracers that passed through monolayers to the BL side were quantified. The transport experiments of TMS-NLCs across MDCK-chAbcg2/abcb1 monolayers were also conducted at 4 °C. To get further insights into the transcellular transport of TMS-NLCs, the monolayers were pre-incubated with various endocytosis inhibitors on the AP side for 1 h and then co-incubated with TMS-NLCs for 4 h. The determination of TMS in the samples collected from the BL side was analyzed by HPLC. 

### 2.8. Antibacterial Activity

Microdilution broth assay was carried out to evaluate the antibacterial activity of API and TMS-NLCs according to the guidelines of the CLSI. The minimal inhibitory concentration (MIC) was determined against 2 strains of Gram-positive bacteria and 2 strains of Gram-negative bacteria. *Staphylococcus aureus* QWJAB25 and *Streptococcus agalactiae* A27 were isolated from milk in a scale farm located in Yangzhou, Jiangsu Province in 2019. The *Escherichia coli* used in this study was ATCC 25922. The *Salmonella Typhimurium* strain 1344 was donated by Associate Professor Jian Lin from College of Life Sciences, Nanjing Agricultural University.

### 2.9. Statistical Analysis

All data shown as mean ± standard deviation (SD) were obtained via at least three independent experiments and analyzed by ANOVA. Statistical analysis was performed using SPSS software under one-way ANOVA. *p* < 0.05 was considered to be statistically significant, and *p* < 0.01 was considered as an extremely significant difference.

## 3. Results and Discussion

### 3.1. The Optimal Preparation Conditions

For oral administration, carriers with particle size less than 300 nm are more suitable for the intestinal transport in order to reduce capture by the reticuloendothelial system [29]. The range of HD values was from 292.50 ± 1.65 nm to 595.30 ± 33.99 nm in the orthogonal design experiment. According to the R values, in which a higher R value represents more significant effect [20], the effect of four factors decreased in the following order: C > B > A > D (Table 2), indicating C, i.e., the ratio of drug to mixed lipids, was the most important effect among these four factors set in this study. In addition, K1, K2, and K3 in Table 2 represented the mean values of evaluation index for the three levels corresponding to one factor. For example, when the factor was A (ratio of stearic acid to oleic acid), the level was 1, and the evaluation index was HD; then, K1 was calculated as follows: K1 = (344.67 + 342.60 + 553.73)/3 = 413.67, which represented the average value of HD for factor A at level 1. The smaller particles would contribute to the intestinal absorption [30], and thus, the K value should be chosen as small as possible. By comparing varied K values, the optimal level combination of factors for HD can be confirmed: A2B2C1D2 (Table 3). The ratio of drug to mixed lipids had a significant impact on HD (*p* < 0.05) (Table S7). It has been reported that particle size has a strong positive relationship with drug and negative relationship with liquid lipid because of its ability to improve molecular mobility and also decrease viscosity [31]. 

**Figure 1:**
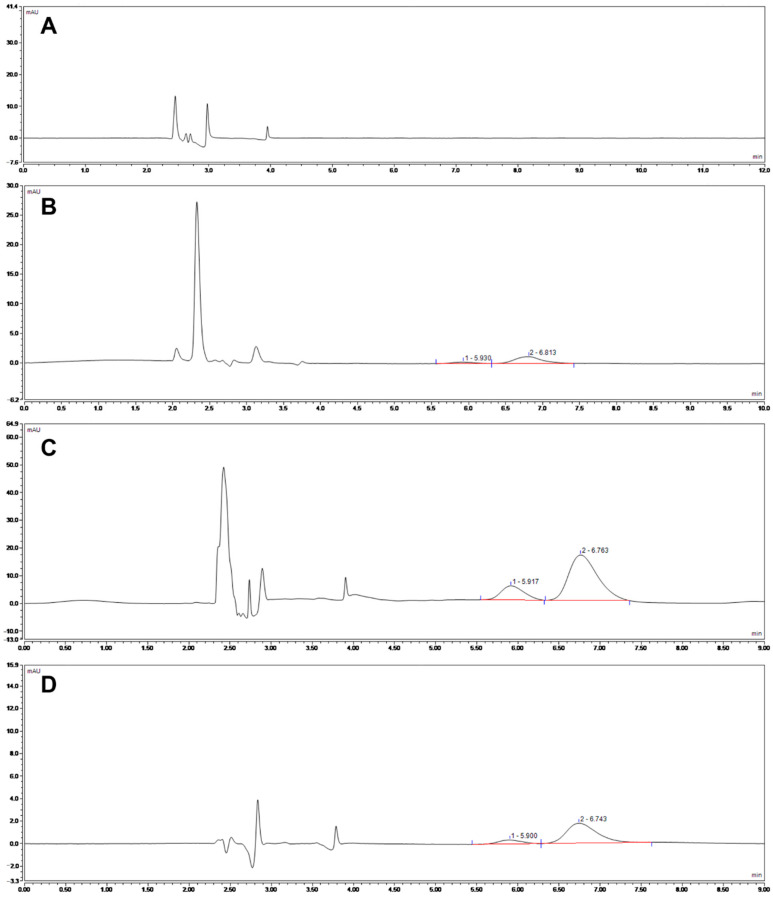
HPLC chromatogram of TMS. (**A**) blank NLCs; (**B**) 10 µg/mL native TMS; (**C**) TMS−NLCs; (**D**) free TMS in filtrate.

According to the R values (Table 2), the effect of four factors for EE decreased in the following order: C > A > B > D, and the optimal level combination for EE was A3B3C3D1 (Table 3). Four factors had no significant impact on EE (*p* > 0.05) (Table S7). All formulations with high EE values above 90% could be ascribed to the addition of liquid lipid, which could cause imperfection in the crystal structure, increased the solubility of TMS, and prevented SA from recrystallizing [32,33]. As shown in Figure 1, the blank NLCs have no influence on the UV spectra of native TMS, and TMS was wrapped in the nanocarrier. The excellent EE suggested that the carriers could efficiently reduce the bitter taste of TMS for oral administration and realize sustained release to improve oral bioavailability [34].

High drug loading is one of the important indexes to evaluate the quality of drug carriers [35]. According to the R values (Table 2), the effect of four factors decreased in the following order: C > B > A > D, the optimal combination for DL was A3B3C3D3 (Table 3). The ratio of drug to mixed lipids had a significant impact on DL (*p* < 0.05), which improved with the increasing TMS content (Table S7). 

### 3.2. Optimization and Characterization of TMS-NLCs

**Figure 2:**
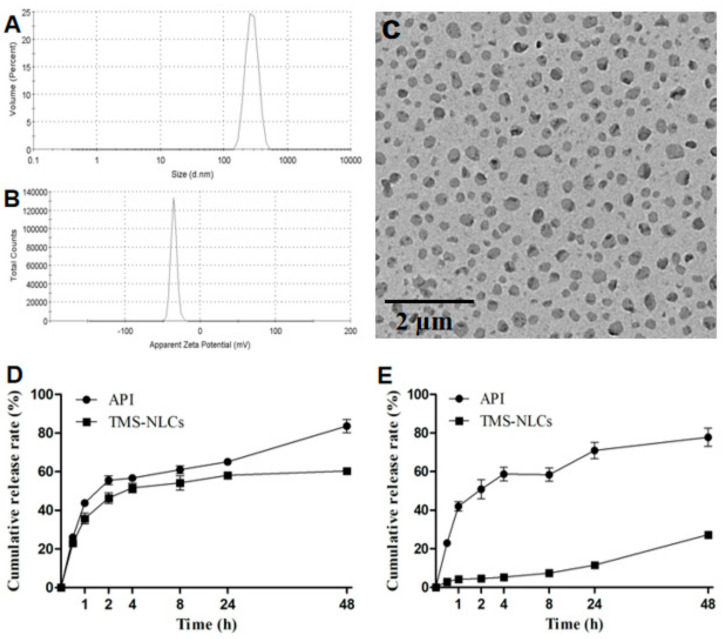
Characterization of the optimized tilmicosin-loaded nanostructured lipid carriers (TMS−NLCs). (**A**,**B**) Hydrodynamic diameter and zeta potential of the optimized TMS−NLCs were determined by dynamic light scattering (DLS). (**C**) The morphology of TMS−NLCs was observed by transmission electron microscopy (TEM). (**D**,**E**) In vitro drug release profiles of TMS−NLCs and active pharmaceutical ingredient (API) were investigated in simulated gastric (pH 1.2) and intestinal fluids (pH 6.8), respectively.

The results of the orthogonal experiment mentioned above indicated that the optimal formulation of the three evaluation indexes was not uniform. As shown in Table S7, the ratio of drug to mixed lipids was the main influencing factor. To obtain a drug carrier with minimum HD and maximum DL, 19 formulations were arranged to further screen the optimized formulation of TMS-NLCs. Herein, the HD, PDI, ZP, EE, and DL of these formulations were characterized (Table 4). The formulation A1-B3-C3-D2 fulfilled the requirements, possessing an HD of 276.85 ± 2.62 nm and DL of 9.14 ± 0.04% (Figure 2A,B), exhibiting smaller HD and higher DL than TMS-HCO-SLNs reported before [13]. Additionally, this formulation had a high EE of 92.92 ± 0.42%, and low PDI of 0.231 ± 0.001 and ZP of −31.10 ± 0.00 mV (Table 4), suggesting that it exhibited the excellent bitterness masking effect and stability in liquid state. The above optimal prescription was used in the follow-up experiment. TEM observation demonstrated that the optimized TMS-NLCs were in approximately spherical shape with uniform dispersion (Figure 2C). According to the analysis of the TEM images, the average size of TMS-NLCs was 188.56 ± 24.45 nm, which was much smaller compared to the HD as determined by DLS due to hydration in the aqueous state [36] and 90% lipid oil in the particle matrix. The in vitro release profiles of TMS-NLCs were evaluated at 37 °C by the dialysis bag method. The release level of API in simulated intestinal fluid (SGF, pH 1.2) and intestinal fluid (SIF, pH 6.8) were similar and approaching 80% in 48 h. However, the release rate of TMS-NLCs was 27% in SIF, and it was lower than 60% in SGF in 48 h (Figure 2D,E). However, there was no significant difference in HD and PDI during 4 h in these two different environments investigated previously by our group [19]. This might be due to the fact that some drugs were distributed on the surface of nanoparticles, and the solubility of tilmicosin in acid medium is higher than that in alkaline medium. On the whole, TMS-NLCs exhibited a slow and sustained release behavior for 48 h in both simulated gastric juice and intestinal fluid in comparison to API, which was perhaps thanks to its high EE for TMS. Furthermore, TMS-NLCs showed an extremely low release level in the simulated saliva (data not shown), suggesting its excellent effect in masking bitterness for TMS [37].

### 3.3. High-Speed Centrifugal Stability 

The stability of nanocarriers is a critical factor for potential biological application [38,39]. Centrifugal stability is a rapid method to detect the stability of the system. After centrifugation at 10,000 rpm, precipitate was seen at the bottom, and a decrease in HD and increase in PDI were also observed, which may be due to nanoparticles coalescence. However, this increase in HD did not seem to be significant, as the K_E_ values were mainly lower than 0.3, and the K_E_ values of TMS-NLCs centrifugation at 4 °C were lower than 25 °C, indicating that this optimized formulation showed high stability and was more suitable to store at 4 °C (Table 5).

### 3.4. Validation of Cell Monolayer Models

#### 3.4.1. Integrity of Cell Monolayers

**Figure 3:**
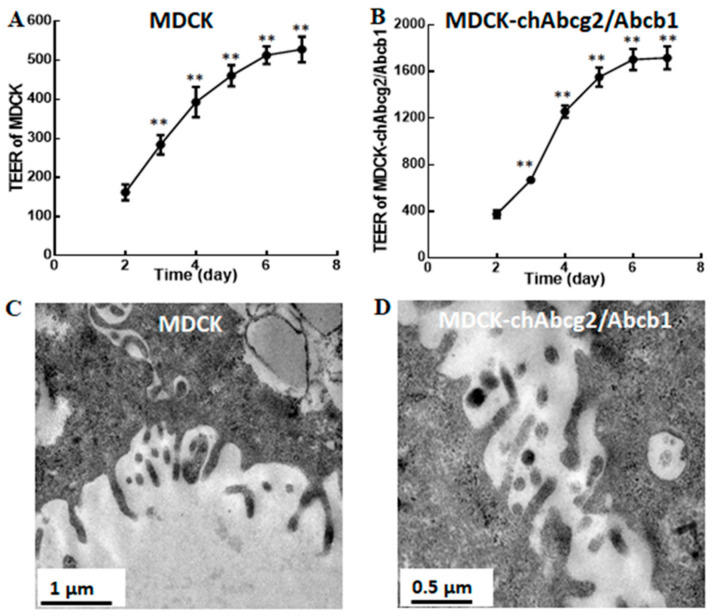
Transepithelial electrical resistance (TEER) and morphology observation of cell monolayers. (**A**,**B**) TEER of Madin–Darby canine kidney (MDCK) and MDCK-chAbcg2/Abcb1 cells monolayers was determined over 6 days. (**C**,**D**) The morphology of MDCK and MDCK-chAbcg2/Abcb1 cells were observed by TEM. ** *p* < 0.01 when compared to the data at Day 2.

The barrier properties of cell monolayers should be evaluated prior to the transmembrane transport experiments. The increasing integrity of cell monolayers is presented as the growing trend of TEER values during the cultivation. TEER monitors the presence of tight junctions (TJs), which regulate paracellular permeation [40]. As shown in Figure 3A,B, the TEER values reached a plateau value 500 Ω·cm^2^ of MDCK and 1700 Ω·cm^2^ of MDCK-chAbcg2/Abcb1 cell monolayers after 7 days, indicating that the integrity of cell monolayers was eligible for follow-up experiments.

#### 3.4.2. Transmission Electron Microscopy of Monolayers

To confirm the degree of cell monolayer differentiation, the cell morphologies were observed by TEM. Although it seemed sparse and stunted, MDCK and MDCK-chAbcg2/Abcb1 cells differentiated into microvilli (Figure 3C,D), mimicking the intestinal epithelium. Based on the above findings, the successfully developed cell monolayers of MDCK and MDCK-chAbcg2/Abcb1 cells could be used for drug transmembrane transport. 

#### 3.4.3. Permeability of Cell Monolayers

The permeability of cell monolayer models is associated with bioavailability in vivo [41]. Lucifer yellow is used as a paracellular permeability marker in epithelial cells. The P_app_ values of lucifer yellow in MDCK and MDCK-chAbcg2/Abcb1 cells were (0.22 ± 0.06) × 10^6^ cm/s and (0.13 ± 0.01) × 10^6^ cm/s, respectively (Table 6), which were less than 0.50 × 10^6^ cm/s specified in the permeability test [42]. Meanwhile, the P_app_ values of propranolol as a transcellular transport marker in MDCK and MDCK-chAbcg2/Abcb1 cells were (16.17 ± 0.65) × 10^6^ cm/s and (17.61 ± 0.64) × 10^6^ cm/s, which were higher than 10 × 10^6^ cm/s according with the requirements of the permeability test [43].

### 3.5. Effect of NLCs and Endocytosis Inhibitors on Cell Viability

**Figure 4:**
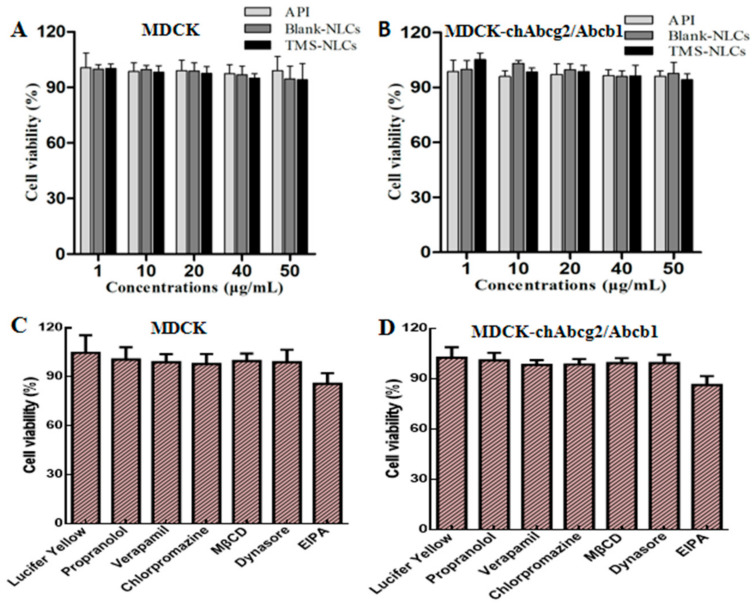
Effect of NLCs, tracers and inhibitors on the viability of MDCK and MDCK-chAbcg2/Abcb1 cells. (**A**,**B**) Cell viability of MDCK and MDCK-chAbcg2/Abcb1 cells treated with API, Blank NLCs and TMS-NLCs after 4h. (**C**,**D**) Cell viability of MDCK and MDCK-chAbcg2/Abcb1 cells treated with tracers and inhibitors.

To screen the non-toxic dose range of drugs to determine the optimal transport concentration of NLCs, MTT assay was applied to evaluate the cell viability. Compared to untreated cells, cell viability was not significantly decreased by API, blank NLCs, and TMS-NLCs when the drug concentrations were up to 50 µg/mL (Figure 4A,B). In the current study, the concentration of API and TMS-NLCs chosen for transport experiments was 10 µg/mL.

The cytotoxicity of endocytosis inhibitors also needed to be evaluated prior to the transport experiments because various cells have different sensitivity to the same drug. The cell viability of all the selected inhibitors was more than 85% on both cells (Figure 4C,D), indicating that the selected inhibitors were available for further transport experiments.

### 3.6. The Permeability of TMS-NLCs across MDCK-chAbcg2/Abcb1 Cell Monolayers

Bidirectional transmembrane transport of drug was estimated to understand whether NLCs could improve the permeability of TMS in MDCK-chAbcg2/Abcb1 expressing P-gp and BCRP [21]. Both transporters belong to ATP-binding cassette transporters class and are known to influence the toxicity and pharmacokinetics of substrate drugs [44]. The NER of API in MDCK-chAbcg2/Abcb1 monolayers was 2.07 (Table 7), which was higher than 2 from the FDA regulations [45], suggesting TMS is a substrate of P-gp. In addition, the NER of API decreasing to 0.94 in the presence of verapamil further confirmed this result. Remarkably, the NER of TMS-NLCs was 1.51, and the P_app_ value of AP→BL for TMS increased significantly, indicating that this carrier can improve the permeability of TMS and also restrain the efflux function of P-gp in MDCK-chAbcg2/Abcb1 cell monolayers. The permeability of drugs in cell monolayers is available to estimate the bioavailability in vivo [46]. These data indicated that the NLCs could improve the intestinal absorption of TMS.

### 3.7. Transport Mechanism of TMS-NLCs across MDCK-chAbcg2/Abcb1 Cell Monolayers

**Figure 5:**
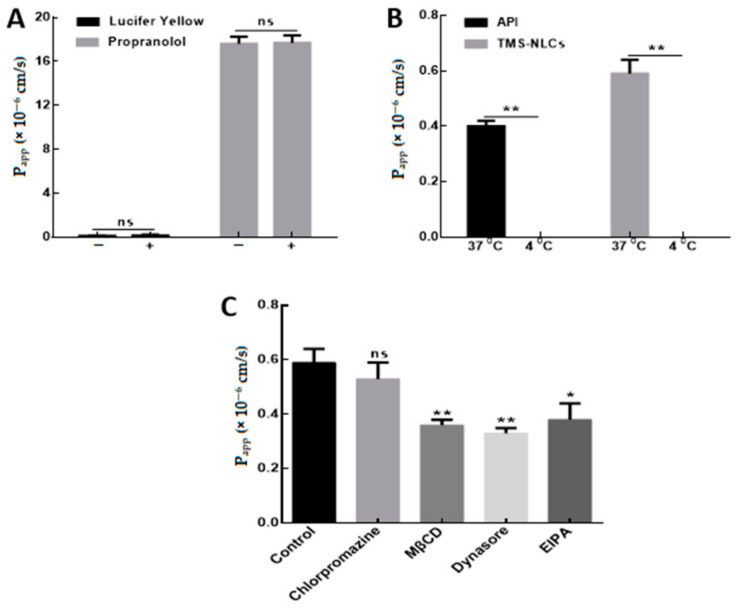
Transport mechanism of TMS-NLCs across MDCK-chAbcg2/Abcb1 cell monolayers. (**A**) The P_app_ of lucifer yellow and propranolol was detected in cell monolayers treated with/without TMS-NLCs, respectively. (**B**) Influence of temperature on the P_app_ of API and TMS-NLCs was determined. (**C**) The P_app_ values of TMS-NLCs were evaluated across cell monolayers pretreated with different endocytosis inhibitors. MβCD: methyl-β-cyclodextrin, EIPA: 5-(*N*-ethyl-*N*-isopropyl)-amiloride; ns: no significance, * *p* < 0.05 and ** *p* < 0.01 when compared to the control except the specific marks.

The P_app_ values of lucifer yellow and propranolol showed no significant difference whether co-incubating with NLCs or not (Figure 5A), indicating that both paracellular and transcellular pathways related to the process of TMS-NLCs across MDCK-chAbcg2/Abcb1 cell monolayers. However, in Caco-2 cell monolayers, the transport pathway of TMS-NLCs was mainly through transcellular pathway in our previous study [19]. In addition, drug transport experiments were respectively conducted at 4 and 37 °C in order to discover whether endocytosis and/or passive mechanisms were involved in the uptake of NLCs. The P_app_ values of API and TMS-NLCs at 4 °C (under the detection limit) were far below 37 °C (Figure 5B), demonstrating that the cellular uptake of TMS-NLCs was an energy-dependent active endocytosis process.

To illuminate the possible cellular internalization pathways of TMS-NLCs, the cells were pretreated with various inhibitors to investigate the interaction between TMS-NLCs and cell membranes. As shown in Figure 5C, the P_app_ values showed no change in the presence of chlorpromazine, which is an inhibitor of clathrin-mediated endocytosis. 5-(*N*-Ethyl-*N*-isopropyl)-amiloride (EIPA), an inhibitor of macropinocytosis, reduced the P_app_ values. Moreover, when the monolayers were co-incubated with MβCD and Dynasore, the inhibitor of caveolae/lipid raft-mediated transport process, the P_app_ values decreased significantly. According to these, TMS-NLCs could be mainly internalized through the caveolae/lipid raft-mediated endocytosis pathway, and the minority might be transported via macropinocytosis, which was not involved in Caco-2 cells [19].

### 3.8. Antibacterial Activity

As shown in Table 8, API and TMS-NLCs exhibited the same MIC values, indicating that the antibacterial activity of the drug was not changed by the preparation and release process. It is reported that the MIC values of suspensions reduce to the same level with decreasing the particle size as native drug, suggesting that TMS-NLCs enhanced the antibacterial activity of tilmicosin, since the drug is only partially released from the nanoparticles considering in vitro drug release property [47]. 

## 4. Conclusions

TMS-NLCs were prepared with a high shear-ultrasound technique and optimized with an orthogonal experiment design. The optimized TMS-NLCs had 276.85 ± 2.62 nm of hydrodynamic diameter, 0.231 ± 0.001 of PDI, −31.10 ± 0.00 mV of zeta potential, 9.14 ± 0.04% of drug loading, and 92.92 ± 0.42% of entrapment efficiency. TMS-NLCs were approximately spherical and exhibited a slow and sustained release behavior without an initial burst release in both simulated gastric juice and intestinal fluid. TMS-NLCs were internalized mainly through the caveolae/lipid raft-mediated endocytosis pathway, and the minority might be transported through macropinocytosis. It was further confirmed that NLCs was available to enhance the permeability of TMS and inhibit the efflux of P-gp in MDCK-chAbcg2/Abcb1 cells. Furthermore, TMS-NLCs had the same antibacterial activity as free TMS. To conclude, it is reasonable to state that NLCs can be an efficient carrier to load TMS for overcoming its oral administration obstacle.

## Figures and Tables

**Table 1:** Factors and levels of orthogonal experiment design.

Levels	Factors			
	A (g/g)	B (wt %)	C (wt %)	D (mL/mL)
1	1:9	20	10	2:1
2	1:6	25	20	1:1
3	1:3	30	30	1:2

Note: A: Ratio of stearic acid and oleic acid. B: Ratio of emulsifier to mixed lipids. C: Ratio of drug to mixed lipids. D: Ratio of cold water to hot emulsion.

**Table 2:** Results and visual analysis of orthogonal L_9_ (3^4^) experiment design.

No.	A (g/g)	B (wt %)	C (wt %)	D (mL/mL)	HD (nm)	EE (%)	DL (%)
1	1	1	1	1	344.67 ± 18.50	90.39 ± 0.092	2.68 ± 0.03
2	1	2	2	2	342.60 ± 10.94	90.02 ± 0.64	6.08 ± 0.04
3	1	3	3	3	553.73 ± 25.37	93.60 ± 0.3	9.49 ± 0.03
4	2	1	2	3	405.67 ± 13.65	90.69 ± 0.67	5.24 ± 0.04
5	2	2	3	1	534.47 ± 29.11	95.00 ± 0.13	8.56 ± 0.01
6	2	3	1	2	292.50 ± 1.65	91.59 ± 0.39	3.39 ± 0.01
7	3	1	3	2	595.30 ± 33.99	93.74 ± 0.52	8.54 ± 0.05
8	3	2	1	3	346.60 ± 5.90	91.72 ± 0.30	3.60 ± 0.01
9	3	3	2	1	407.57 ± 32.38	94.26 ± 0.24	6.38 ± 0.02
HD							
K1	413.67	448.55	327.92	428.90			
K2	410.88	407.89	385.28	410.13			
K3	449.82	417.93	561.17	435.33			
R	38.94	40.66	233.24	25.20			
EE							
K1	91.34	91.61	91.23	93.22			
K2	92.43	92.25	91.66	91.78			
K3	93.24	93.15	94.11	92.00			
R	1.90	1.54	2.88	1.43			
DL							
K1	6.08	5.49	3.22	5.87			
K2	5.73	6.08	5.90	6.00			
K3	6.17	6.42	8.86	6.11			
R	0.44	0.93	5.64	0.24			

Note: HD: hydrodynamic diameter; EE: encapsulation efficiency; DL: drug loading.

**Table 3:** Analysis results of orthogonal experiment design.

Evaluating Indicator	Primary and Secondary Order	Optimal Formulations
HD	C > B > A > D	A2B2C1D2
EE	C > A > B > D	A3B3C3D1
DL	C > B > A > D	A3B3C3D3

**Table 4:** Characterization summary about all formulations in orthogonal optimization experiments.

No.	Formulations	HD (nm)	PDI	ZP (mV)	EE (%)	DL (%)
1	A1B3C1D1	283.70 ± 4.10	0.249 ± 0.059	−32.75 ± 1.63	89.21 ± 1.10	3.04 ± 0.04
2	A1B3C1D2	273.30 ± 10.04	0.288 ± 0.071	−32.90 ± 1.84	91.50 ± 1.65	3.38 ± 0.06
3	A1B2C2D1	292.15 ± 22.42	0.192 ± 0.031	−33.50 ± 0.57	88.50 ± 0.96	4.69 ± 0.05
4	A1B2C2D3	281.60 ± 6.22	0.337 ± 0.033	−33.70 ± 0.71	87.56 ± 0.33	5.60 ± 0.02
5	A1B3C2D1	277.10 ± 11.31	0.264 ± 0.067	−31.10 ± 0.99	91.26 ± 0.21	6.71 ± 0.02
6	A1B3C2D2	264.60 ± 10.32	0.291 ± 0.011	−33.70 ± 0.42	89.54 ± 1.09	5.57 ± 0.07
7	A1B3C2D3	272.40 ± 2.55	0.339 ± 0.023	−33.65 ± 0.21	88.05 ± 0.94	6.75 ± 0.07
8	A1B3C3D1	268.20 ± 9.19	0.239 ± 0.059	−29.60 ± 0.99	92.51 ± 0.08	8.66 ± 0.01
9	A1B3C3D2	276.85 ± 2.62	0.231 ± 0.001	−31.10 ± 0.00	92.92 ± 0.42	9.14 ± 0.04
10	A1B3C3D3	279.10 ± 2.40	0.252 ± 0.042	−30.65 ± 0.35	87.01 ± 1.02	8.20 ± 0.10
11	A3B3C2D1	291.05 ± 0.07	0.232 ± 0.001	−29.25 ± 1.06	92.94 ± 0.62	7.25 ± 0.05
12	A3B3C2D2	306.30 ± 4.81	0.268 ± 0.000	−29.60 ± 0.14	90.11 ± 1.05	6.54 ± 0.08
13	A3B3C2D3	309.65 ± 3.18	0.260 ± 0.007	−30.20 ± 0.14	87.42 ± 0.23	6.40 ± 0.02
14	A3B3C3D1	336.75 ± 13.51	0.238 ± 0.081	−29.20 ± 2.26	93.93 ± 0.62	9.51 ± 0.06
15	A3B3C3D2	316.30 ± 2.69	0.310 ± 0.042	−29.80 ± 1.41	90.13 ± 1.03	8.15 ± 0.09
16	A2B2C1D2	305.79 ± 16.18	0.290 ± 0.019	−31.49 ± 0.86	96.78 ± 0.63	2.77 ± 0.19
17	A1B3C1D3	294.02 ± 11.29	0.372 ± 0.023	−31.27 ± 0.57	94.59 ± 0.57	2.49 ± 0.06
18	A1B2C2D2	362.20 ± 8.64	0.267 ± 0.008	−32.51 ± 0.37	96.41 ± 0.19	5.45 ± 0.15
19	A3B3C3D3	449.61 ± 5.78	0.391 ± 0.055	−27.77 ± 0.72	91.68 ± 0.55	8.56 ± 0.17

Note: HD: hydrodynamic diameter; PDI: polydispersity index; ZP: zeta potential; EE: encapsulation efficiency; DL: drug loading.

**Table 5:** The high-speed centrifugal stability of TMS-NLCs.

Conditions		HD (nm)	PDI	ZP (mV)	K_E_	Precipitate
Untreatment		278.93 ± 5.59	0.215 ± 0.035	−30.20 ± 0.89	-	-
4 °C	5 min	219.30 ± 4.24	0.251 ± 0.008	−30.10 ± 1.27	0.212 ± 0.014	-
	10 min	219.05 ± 2.19	0.270 ± 0.042	−29.70 ± 1.13	0.213 ± 0.007	-
	20 min	198.50 ± 2.12	0.384 ± 0.096	−31.05 ± 0.35	0.286 ± 0.007	+
	30 min	194.70 ± 2.26	0.198 ± 0.002	−29.95 ± 0.21	0.299 ± 0.007	+
25 °C	5 min	196.60 ± 4.81	0.365 ± 0.033	−29.05 ± 0.35	0.292 ± 0.016	-
	10 min	193.95 ± 15.20	0.399 ± 0.062	−30.50 ± 0.57	0.304 ± 0.049	+
	20 min	193.70 ± 11.46	0.254 ± 0.037	−28.90 ± 0.57	0.302 ± 0.037	+
	30 min	200.90 ± 7.07	0.277 ± 0.049	−29.20 ± 0.99	0.278 ± 0.023	+

**Table 6:** Apparent permeability (P_app_) values of lucifer yellow and propranolol transport across MDCK and MDCK-chAbcg2/Abcb1 cell monolayers.

Tracers	Concentration (µM)	P_app_ (×10^−6^ cm/s)	
		MDCK	MDCK-chAbcg2/Abcb1
Lucifer yellow	225	0.22 ± 0.06	0.13 ± 0.01
Propranolol	100	16.17 ± 0.65	17.61 ± 0.64

**Table 7:** Apparent permeability (P_app_), efflux rate (ER), and net efflux rate (NER) of API and TMS-NLCs transport into MDCK and MDCK-chAbcg2/Abcb1 cell monolayers.

Formulations	Verapamil	MDCK			MDCK-chAbcg2/Abcb1			NER
		P_app_ (×10^−6^ cm/s)		ER	P_app_ (×10^−6^ cm/s)		ER	
		AP→BL	BL→AP		AP→BL	BL→AP		
API	−	0.42 ± 0.04	0.53 ± 0.02	1.27	0.40 ± 0.02	1.06 ± 0.03	2.63	2.07
	+	0.38 ± 0.02	0.41 ± 0.04	1.06	0.39 ± 0.04	0.39 ± 0.07 **	1.00	0.94
TMS-NLCs	−	0.48 ± 0.01	0.51 ± 0.02	1.07	0.59 ± 0.05 **	0.96 ± 0.11	1.62	1.51
	+	0.49 ± 0.06	0.47 ± 0.05	0.96	0.36 ± 0.04	0.36 ± 0.08 **	1.00	1.04

Note: −: treatment without verapamil; +: treatment with verapamil; ** *p* < 0.01 represented extremely significant difference marked by **.

**Table 8:** Minimal inhibitory concentration (MIC) (µg/mL) of API and TMS-NLCs.

Formulations	Strains			
	*S. aureus*	*S. agalactiae*	*E. coli*	*S. Typhimurium*
API	<0.5	16	16	16
TMS-NLCs	<0.5	16	16	16

## Data Availability

Data is contained within the article or supplementary material.

## Supplementary Materials

The following are available online at https://www.mdpi.com/1999-4923/13/3/303/s1, Table S1: Effect of emulsifier to lipid ratio (ELR) on the hydrodynamic diameters (HD), polydispersity index (PDI) and zeta potentials (ZP) of TMS-NLCs (n = 3), Table S2: Effect of SA to OA ratio (SOR) on the hydrodynamic diameters (HD), polydispersity index (PDI) and zeta potentials (ZP) of TMS-NLCs (n = 3), Table S3: Effect of drug to lipid ratio (DLR) on the hydrodynamic diameters (HD), polydispersity index (PDI) and zeta potentials (ZP) of TMS-NLCs (n = 3), Table S4: Effect of cold water dispersion volume (CWDV) on the hydrodynamic diameters (HD), polydispersity index (PDI) and zeta potentials (ZP) of TMS-NLCs (n = 3), Table S5: Effect of ultrasonic time (UT) on the hydrodynamic diameters (HD), polydispersity index (PDI) and zeta potentials (ZP) of TMS-NLCs (n = 3), Table S6: Effect of ultrasonic time (UT) on the hydrodynamic diameters (HD), polydispersity index (PDI) and zeta potentials (ZP) of TMS-NLCs (n = 3), Table S7: Analysis of variance.

## References

[1] Haider M., Abdin S.M., Kamal L., Orive G. (2020). Nanostructured lipid carriers for delivery of chemotherapeutics: A review. Pharmaceutics.

[2] Pucek A., Tokarek B., Waglewska E., Bazylińska U. (2020). Recent advances in the structural design of photosensitive agent formulations using “soft” colloidal nanocarriers. Pharmaceutics.

[3] Chen C., Lee Y., Chang S., Tsai Y., Fang J., Hwang T. (2019). Oleic acid-loaded nanostructured lipid carrier inhibit neutrophil activities in the presence of albumin and alleviates skin inflammation. Int. J. Nanomed..

[4] Jnaidi R., Almeida A.J., Gonçalves L.M. (2020). Solid lipid nanoparticles and nanostructured lipid carriers as smart drug delivery systems in the treatment of Glioblastoma Multiforme. Pharmaceutics.

[5] McClements D.J. (2020). Advances in nanoparticle and microparticle delivery systems for increasing the dispersibility, stability, and bioactivity of phytochemicals. Biotechnol. Adv..

[6] Garcês A., Amaral M.H., Sousa Lobo J.M., Silva A.C. (2018). Formulations based on solid lipid nanoparticles (SLN) and nanostructured lipid carriers (NLC) for cutaneous use: A review. Eur. J. Pharm. Sci..

[7] Beloqui A., Solinís M.Á., Rodríguez-Gascón A., Almeida A.J., Préat V. (2016). Nanostructured lipid carriers: Promising drug delivery systems for future clinics. Nanomed Nanotechnol..

[8] Gaba B., Fazil M., Ali A., Baboota S., Sahni J.K., Ali J. (2015). Nanostructured lipid (NLCs) carriers as a bioavailability enhancement tool for oral administration. Drug Deliv..

[9] Ziv G., Shem-Tov M., Glickman A., Winkler M., Saran A. (1995). Tilmicosin antibacterial activity and pharmacokinetics in cows. J. Vet. Pharmacol. Ther..

[10] IbrahimA E., Abdel-Daim M.M. (2015). Modulating effects of Spirulina platensis against tilmicosin-induced cardiotoxicity in mice. Cell J..

[11] Al-Qushawi A., Rassouli A., Atyabi F., Peighambari S.M., Esfandyari-Manesh M., Shams G.R., Yazdani A. (2016). Preparation and characterization of three tilmicosin-loaded lipid nanoparticles: Physicochemical properties and in-vitro antibacterial activities. Iran. J. Pharm. Res..

[12] Vogel G.J., Laudert S.B., Zimmermann A., Guthrie C.A., Mechor G.D., Moore G.M. (1998). Effects of tilmicosin on acute undifferentiated respiratory tract disease in newly arrived feedlot cattle. JAVMA J. Am. Vet. Med. A.

[13] Xie S., Wang F., Wang Y., Zhu L., Dong Z., Wang X., Li X., Zhou W. (2011). Acute toxicity study of tilmicosin-loaded hydrogenated castor oil-solid lipid nanoparticles. Part. Fibre. Toxicol..

[14] Xiong J., Zhu Q., Zhao Y., Yang S., Cao J., Qiu Y. (2019). Tilmicosin enteric granules and premix to pigs: Antimicrobial susceptibility testing and comparative pharmacokinetics. J. Vet. Pharmacol. Ther..

[15] Song M., Li Y., Fai C., Cui S., Cui B. (2011). The controlled release of tilmicosin from silica nanoparticles. Drug Dev. Ind. Pharm..

[16] Zhou K., Wang X., Chen D., Yuan Y., Wang S., Li C., Yan Y., Liu Q., Shao L., Huang L. (2019). Enhanced treatment effects of tilmicosin against Staphylococcus aureus cow mastitis by self-assembly sodium alginate-chitosan nanogel. Pharmaceutics.

[17] Yu J., Wang M., Bhutto R.A., Zhao H., Cohen Stuart M.A., Wang J. (2020). Facile preparation of tilmicosin-loaded polymeric nanoparticle with controlled properties and functions. ACS Omega.

[18] Sahito B., Zhang Q., Yang H., Peng L., Gao X., Kashif J., Aabdin Z.U., Jiang S., Wang L., Guo D. (2020). Synthesis of tilmicosin nanostructured lipid carriers for improved oral delivery in broilers: Physiochemical characterization and cellular permeation. Molecules.

[19] Zhang Q., Yang H., Sahito B., Li X., Peng L., Gao X., Ji H., Wang L., Jiang S., Guo D. (2020). Nanostructured lipid carriers with exceptional gastrointestinal stability and inhibition of P-gp efflux for improved oral delivery of tilmicosin. Colloid Surf. B.

[20] Dai C., Zhang Z., Wang T. (2019). Preparation and heat-insulating properties of Al2O3–ZrO2 (Y2O3) hollow fibers derived from cogon using an orthogonal experimental design. RSC Adv..

[21] Zhang Y., Huang J., Liu Y., Guo T., Wang L. (2018). Using the lentiviral vector system to stably express chicken P-gp and BCRP in MDCK cells for screening the substrates and studying the interplay of both transporters. Arch. Toxicol..

[22] Zhang Q., Sahito B., Li L., Peng L., Jiang S., Guo D. (2019). Determination of encapsulation efficacy and loading capacity of tilmicosin loaded nanostructured lipid carriers by ultrafiltration centrifugation combined with high performance liquid chromatography. Anim. Hus. Vet. Med..

[23] Neves A.R., Queiroz J.F., Lima S.A.C., Figueiredo F., Fernandes R., Reis S. (2016). Cellular uptake and transcytosis of lipid-based nanoparticles across the intestinal barrier: Relevance for oral drug delivery. J. Colloid Interf. Sci..

[24] Beloqui A., Solinís M.Á., Gascón A.R., Pozo-Rodríguez A.D., Rieux A.D., Préat V. (2013). Mechanism of transport of saquinavir-loaded nanostructured lipid carriers across the intestinal barrier. J. Control Release.

[25] He B., Jia Z., Du W., Yu C., Fan Y., Dai W., Yuan L., Zhang H., Wang X., Wang J. (2013). The transport pathways of polymer nanoparticles in MDCK epithelial cells. Biomaterials.

[26] Wassermann L., Halwachs S., Lindner S., Honscha K.U., Honscha W. (2013). Determination of functional ABCG2 activity and assessment of drug-ABCG2 interactions in dairy animals using a novel MDCKII in vitro model. J. Pharm. Sci..

[27] Roger E., Lagarce F., Garcion E., Benoit J.P. (2009). Lipid nanocarriers improve paclitaxel transport throughout human intestinal epithelial cells by using vesicle-mediated transcytosis. J. Control Release.

[28] Chai G., Xu Y., Chen S., Cheng B., Hu F., You J., Du Y., Yuan H. (2016). Transport mechanisms of solid lipid nanoparticles across Caco-2 cell monolayers and their related cytotoxicology. ACS Appl. Mater. Inter..

[29] Singh I., Swami R., Khan W., Sistla R. (2014). Lymphatic system: A prospective area for advanced targeting of particulate drug carriers. Expert Opin. Drug Del..

[30] He Z., Hu Y., Nie T., Tang H., Zhu J., Chen K., Liu L., Leong K.W., Chen Y., Mao H. (2018). Size-controlled lipid nanoparticle production using turbulent mixing to enhance oral DNA delivery. Acta Biomater..

[31] Chen C., Tsai T., Huang Z., Fang J. (2010). Effects of lipophilic emulsifiers on the oral administration of lovastatin from nanostructured lipid carriers: Physicochemical characterization and pharmacokinetics. Eur. J. Pharm. Biopharm..

[32] Tiwari R., Pathak K. (2011). Nanostructured lipid carrier versus solid lipid nanoparticles of simvastatin: Comparative analysis of characteristics, pharmacokinetics and tissue uptake. Int. J. Pharmaceut..

[33] ElShaer A., Mustafa S., Kasar M., Thapa S., Ghatora B., Alany R.G. (2016). Nanoparticle-laden contact lens for controlled ocular delivery of prednisolone: Formulation optimization using statistical experimental design. Pharmaceutics.

[34] Zhang Y., Shen L., Wang T., Li H., Huang R., Zhang Z., Wang Y., Quan D. (2020). Taste masking of water-soluble drug by solid lipid microspheres: A child-friendly system established by reversed lipid-based nanoparticle technique. J. Pharm. Pharmacol..

[35] Wang T., Xue J., Hu Q., Zhou M., Luo Y. (2017). Preparation of lipid nanoparticles with high loading capacity and exceptional gastrointestinal stability for potential oral delivery applications. J. Colloid Interf. Sci..

[36] Mehnert W., Karsten M. (2012). Solid lipid nanoparticles: Production, characterization and applications. Adv. Drug Deliver. Rev..

[37] Bazylińska U., Kulbacka J., Chodaczek G. (2019). Nanoemulsion Structural Design in Co-Encapsulation of Hybrid Multifunctional Agents: Influence of the Smart PLGA Polymers on the Nanosystem-Enhanced Delivery and Electro-Photodynamic Treatment. Pharmaceutics.

[38] Shakeel F., Ramadan W. (2009). Transdermal delivery of anticancer drug caffeine from water-in-oil nanoemulsions. Colloid Surface B.

[39] Yan G., Liang Q., Wen X., Peng J., Deng R., Lv L., Ji M., Deng X., Wu L., Feng X. (2020). Preparation, characterization, and pharmacokinetics of tilmicosin taste-masked formulation via hot-melt extrusion technology. Colloid Surf. B.

[40] Ayehunie S., Islam A., Cannon C., Landry T., Pudney J., Klausner M., Anderson D.J. (2015). Characterization of a hormone-responsive organotypic human vaginal tissue model: Morphologic and immunologic effects. Reprod. Sci..

[41] Gamboa J.M., Leong K.W. (2013). In vitro and in vivo models for the study of oral delivery of nanoparticles. Adv. Drug Deliver. Rev..

[42] Cho M.J., Thompson D.P., Cramer C.T., Vidmar T.J., Scieszka J.F. (1989). The Madin Darby canine kidney (MDCK) epithelial cell monolayer as a model cellular transport barrier. Pharm. Res..

[43] Yang X., Yang X., Wang Y., Ma L., Zhang Y., Yang X., Wang K. (2007). Establishment of Caco-2 cell monolayer model and standard operation procedure for assessing intestinal absorption of chemical components of traditional Chinese medicine. J. Chin. Integr. Med..

[44] Choi Y.H., Yu A. (2014). ABC transporters in multidrug resistance and pharmacokinetics, and strategies for drug development. Curr. Pharm. Des..

[45] National Archives & Records Service of Office (2012). FDA draft guidance for industry on drug interaction studies-study design, data analysis, implications for dosing, and labeling recommendations. Availab. Fed. Regist..

[46] Ma B., Wang J., Sun J., Li M., Xu H., Sun G., Sun X. (2014). Permeability of rhynchophylline across human intestinal cell in vitro. Int. J. Clin. Exp. Patho..

[47] Chen X., Wang T., Lu M., Zhu L., Wang Y., Zhou W. (2014). Preparation and evaluation of tilmicosin-loaded hydrogenated castor oil nanoparticle suspensions of different particle sizes. Int. J. Nanomed..

