# Supplementary material for: Optimization of Tilmicosin-Loaded Nanostructured Lipid Carriers Using Orthogonal Design for Overcoming Oral Administration Obstacle
Source: Pharmaceutics. 2021 Feb 25;13(3):303. doi: 10.3390/pharmaceutics13030303 (PMC7996536; doi:10.3390/pharmaceutics13030303)
Supplement: Supplementary file 1 [file pharmaceutics-13-00303-s001.pdf]

pharmaceutics

Supplementary Materials: Optimization of Tilmicosin-Loaded Nanostructured Lipid Carriers Using Orthogonal Design for Overcoming Oral Administration Obstacle

Jia Wen [1,†], Xiuge Gao [1,†], Qian Zhang [1], Benazir Sahito [1], Hongbin Si [2], Gonghe Li [2], Qi Ding [3], Wenda Wu [1,4,*], Eugenie Nepovimova [4], Shanxiang Jiang [1], Liping Wang [1], Kamil Kuca [4,*] and Dawei Guo [1,*]

1. Results of single factor experiments

1.1. Effect of emulsifier to lipid ratio on TMS-NLCs

It was firstly evaluated the effect of emulsifier to lipid ratio on the hydrodynamic diameters (HD), polydispersity index (PDI) and zeta potentials (ZP) of TMS-NLCs. Herein, emulsifier to lipid ratio was set in the range from 5% to 60%. Other parameters were fitted to 1:9 of SA to OA ratio, 10% of drug to lipid ratio, 10 mL of water phrase, and 11 mL of cold water dispersion volume. As shown in Table S1, HD of TMS-NLCs gradually decreased with increased amounts of emulsifiers, all PDIs were close to 0.3, and ZPs were ranging from −36 mV to −40 mV. In order to use emulsifiers as little as possible, three levels of 20%, 25% and 30% were used in the later orthogonal test.

Table S1. Effect of emulsifier to lipid ratio (ELR) on the hydrodynamic diameters (HD), polydispersity index (PDI) and zeta potentials (ZP) of TMS-NLCs (n=3).

ELR (%)	HD (nm)	PDI	ZP (mV)
**5**	434.25 ± 17.32	0.329 ± 0.047	−39.45 ± 1.06
**10**	329.95 ± 9.97	0.372 ± 0.036	−38.60 ± 0.71
**15**	375.30 ± 18.38	0.199 ± 0.033	−39.45 ± 0.35
**20**	288.70 ± 3.10	0.294 ± 0.028	−35.20 ± 1.49
**25**	285.45 ± 3.32	0.264 ± 0.025	−39.30 ± 0.85
**30**	243.75 ± 5.02	0.323 ± 0.003	−38.65 ± 0.92
**40**	243.90 ± 6.65	0.284 ± 0.044	−37.35 ± 0.78
**50**	227.90 ± 3.39	0.305 ± 0.042	−36.90 ± 0.42
**60**	218.60 ± 4.24	0.337 ± 0.008	−36.60 ± 0.57

Publisher's Note: MDPI stays neutral with regard to jurisdictional claims in published maps and institutional affiliations.

Copyright: © 2021 by the author. Licensee MDPI, Basel, Switzerland. This article is an open access article distributed under the terms and conditions of the Creative Commons Attribution (CC BY) license (http://creativecommons.org/licenses/by/4.0/).

1.2. Effect of SA to OA ratio on TMS-NLCs

The HD, PDI and ZP of TMS-NLCs were studied when SA to OA ratio is set to 1/9, 1/8, 1/7, 1/6, 1/5, 1/4, 1/3, 3/7, 1/2, 2/3, and 1/1, respectively. Other parameters were fitted to 25% of emulsifier to lipid ratio, 10% of drug to lipid ratio, 10 mL of water phrase, and 11 mL of cold water dispersion volume. As shown in Table S2, HD of TMS-NLCs gradually increased with enhanced SA to OA ratio, especially from 1/3 to 1/1. Therefore, 1/9, 1/6 and 1/3 were selected as the three levels in the orthogonal test.

Table S2. Effect of SA to OA ratio (SOR) on the hydrodynamic diameters (HD), polydispersity index (PDI) and zeta potentials (ZP) of TMS-NLCs (*n*=3).

SOR (g/g)	HD (nm)	PDI	ZP (mV)
1/9	311.50 ± 15.84	0.278 ± 0.007	−34.50 ± 0.57
1/8	332.65 ± 19.16	0.381 ± 0.021	−35.40 ± 0.28
1/7	355.95 ± 12.09	0.429 ± 0.021	−36.10 ± 1.13
1/6	335.45 ± 1.20	0.310 ± 0.062	−31.15 ± 2.05
1/5	355.55 ± 0.07	0.311 ± 0.008	−30.95 ± 1.06
1/4	345.75 ± 1.63	0.244 ± 0.074	−30.30 ± 1.27
1/3	355.05 ± 11.53	0.303 ± 0.008	−30.50 ± 0.85
3/7	410.35 ± 4.74	0.154 ± 0.070	−28.90 ± 0.85
1/2	396.45 ± 15.77	0.303 ± 0.016	−30.50 ± 1.41
2/3	432.55 ± 3.61	0.260 ± 0.013	−27.05 ± 0.92
1/1	495.30 ± 1.84	0.243 ± 0.043	−25.25 ± 0.07

1.3. Effect of drug to mixed lipid ratio on TMS-NLCs

It has been reported that the addition amounts of drug would influence the parameters of TMS-NLCs, especially HD. In this study, we investigated that effect of drug to lipid ratio on the parameters of TMS-NLCs. Herein, other parameters were fitted to 1:3 of SA to OA ratio, 25% of emulsifier to lipid ratio, 10% of drug to lipid ratio, 10 mL of water phrase, and 11 mL of cold water dispersion volume. The results showed that HD of TMS-NLCs gradually increased when the drug to lipid ratio was enhanced from 5% to 50% while there was no significant difference in ZP and PDI (Table S3). In order to require the small size of TMS-NLCs, 10%, 20%, and 30% was selected as the three levels of drug to lipid ratio.

Table S3. Effect of drug to lipid ratio (DLR) on the hydrodynamic diameters (HD), polydispersity index (PDI) and zeta potentials (ZP) of TMS-NLCs (*n*=3).

DLR (w/w)	HD (nm)	PDI	ZP (mV)
0	289.30 ± 3.54	0.322 ± 0.029	−29.00 ± 0.14
5%	341.80 ± 4.10	0.332 ± 0.011	−30.85 ± 0.49
10%	333.75 ± 2.33	0.420 ± 0.009	−29.65 ± 0.64
15%	391.80 ± 7.92	0.354 ± 0.003	−31.05 ± 0.21

20%	393.50 ± 3.68	0.314 ± 0.050	−28.20 ± 0.71
30%	447.90 ± 2.12	0.375 ± 0.083	−27.70 ± 0.42
40%	548.55 ± 17.18	0.448 ± 0.078	−30.90 ± 0.42
50%	702.05 ± 0.49	0.466 ± 0.025	−30.25 ± 0.21

1.4. Effect of cold water to hot emulsion ratio on TMS-NLCs

Effect of cold water dispersion volume on TMS-NLCs was investigated when other parameters were fitted to 1:3 of SA to OA ratio, 25% of emulsifier to lipid ratio, 10% of drug to lipid ratio and 10 mL of water phrase. As shown in Table S4, the more cold water dispersion volume the bigger HD, while there was no significant changes in ZP and PDI. 2/1, 1/1 and 1/2 was hence chosen as the three levels of cold water to hot emulsion ratio.

Table S4. Effect of cold water dispersion volume (CWDV) on the hydrodynamic diameters (HD), polydispersity index (PDI) and zeta potentials (ZP) of TMS-NLCs (*n*=3).

CWDV	HD (nm)	PDI	ZP (mV)
2/1	337.95 ± 13.08	0.303 ± 0.028	−32.70 ± 0.57
1/1	340.75 ± 10.54	0.328 ± 0.037	−31.40 ± 0.42
1/2	355.00 ± 14.71	0.328 ± 0.034	−29.65 ± 1.06
1/3	391.60 ± 6.08	0.284 ± 0.025	−29.05 ± 0.92

1.5. Effect of ultrasonic time on TMS-NLCs

Effect of ultrasonic time on TMS-NLCs was evaluated when other parameters were fitted to 1:9 of SA to OA ratio, 30% of emulsifier to lipid ratio, 10% of drug to lipid ratio, 10 mL of water phrase and 11 mL of cold water dispersion volume. As exhibited in Table S5, HD of TMS-NLCs would gradually decrease along with the increasing ultrasonic time ranging from 5 min to 20 min, while HD would increase when the ultrasonic time was more than 20 min. Generally, TMS-NLCs was relatively superior in all HD, PDI and ZP when the ultrasonic time was 20 min. Therefore, the ultrasoinc time was fitted to 20 min in the orthogonal experiments.

Table S5. Effect of ultrasonic time (UT) on the hydrodynamic diameters (HD), polydispersity index (PDI) and zeta potentials (ZP) of TMS-NLCs (*n*=3).

UT (min)	HD (nm)	PDI	ZP (mV)
5	306.20 ± 23.48	0.315 ± 0.018	−35.90 ± 0.71
10	266.40 ± 7.07	0.257 ± 0.043	−33.45 ± 0.35
15	299.45 ± 16.33	0.268 ± 0.062	−32.20 ± 0.14
20	235.90 ± 1.41	0.261 ± 0.054	−31.80 ± 1.13
30	258.80 ± 8.63	0.261 ± 0.008	−30.00 ± 0.14

1.6. Effect of cold water dispersion time on TMS-NLCs

Effect of cold water dispersion time on TMS-NLCs was analyzed when other parameters were fitted to 1:9 of SA to OA ratio, 30% of emulsifier to lipid ratio, 10% of drug to

lipid ratio, 10 mL of water phrase and 11 mL of cold water dispersion volume. As presented in Table S6, HD of TMS-NLCs would gradually decreased with the heightened cold water dispersion time ranging from 60s to 120s, while HD would increase when the cold water dispersion time was over 60s. On the whole, TMS-NLCs was relatively superior in HD, PDI and ZP when the cold water dispersion time was 60s. Therefore, the cold water dispersion time was set to 60s in the orthogonal experiments.

Table S6. Effect of cold water dispersion time (CWDT) on the hydrodynamic diameters (HD), polydispersity index (PDI) and zeta potentials (ZP) of TMS-NLCs (*n*=3).

CWDT (s)	HD (nm)	PDI	ZP (mV)
15	290.75 ± 2.86	0.371 ± 0.019	−34.50 ± 1.70
30	300.50 ± 2.19	0.351 ± 0.005	−32.85 ± 0.49
45	266.40 ± 7.07	0.257 ± 0.043	−33.45 ± 0.35
60	244.65 ± 0.39	0.269 ± 0.065	−31.35 ± 0.35
90	327.45 ± 4.91	0.281 ± 0.013	−34.40 ± 1.56
120	258.35 ± 1.17	0.264 ± 0.004	−30.85 ± 1.06

2. Results of Analysis of variance

According to analysis of variance in Table S7, the ratio of drug to mixed lipids had a significant impact on HD (p<0.05). Four factors had no significant impact on EE (p>0.05). The ratio of drug to mixed lipids had a significant impact on DL (p<0.05), which improved with the increasing TMS content.

Table S7. Analysis of variance.

Factor	Sum of squares of deviations	Freedom	F ratio	Significance
HD				
A	2831.65	2	2.75	p>0.05
B	2691.01	2	2.62	p>0.05
C	88628.36	2	86.16*	p<0.05
D	1028.70	2	1.00	p>0.05
Error	1028.70	2		
EE				
A	5.47	2	1.53	p>0.05
B	3.61	2	1.01	p>0.05
C	14.51	2	4.06	p>0.05
D	3.58	2	1.00	p>0.05
Error	3.58	2		
DL				

A	0.33	2	3.92	p>0.05
B	1.34	2	15.94	p>0.05
C	47.76	2	568.51*	p<0.05
D	0.08	2	1.00	p>0.05
Error	0.08	2		

Note: $F_{0.05(2,2)}$ =19.00; $p < 0.05$ represented significant difference marked by *.
